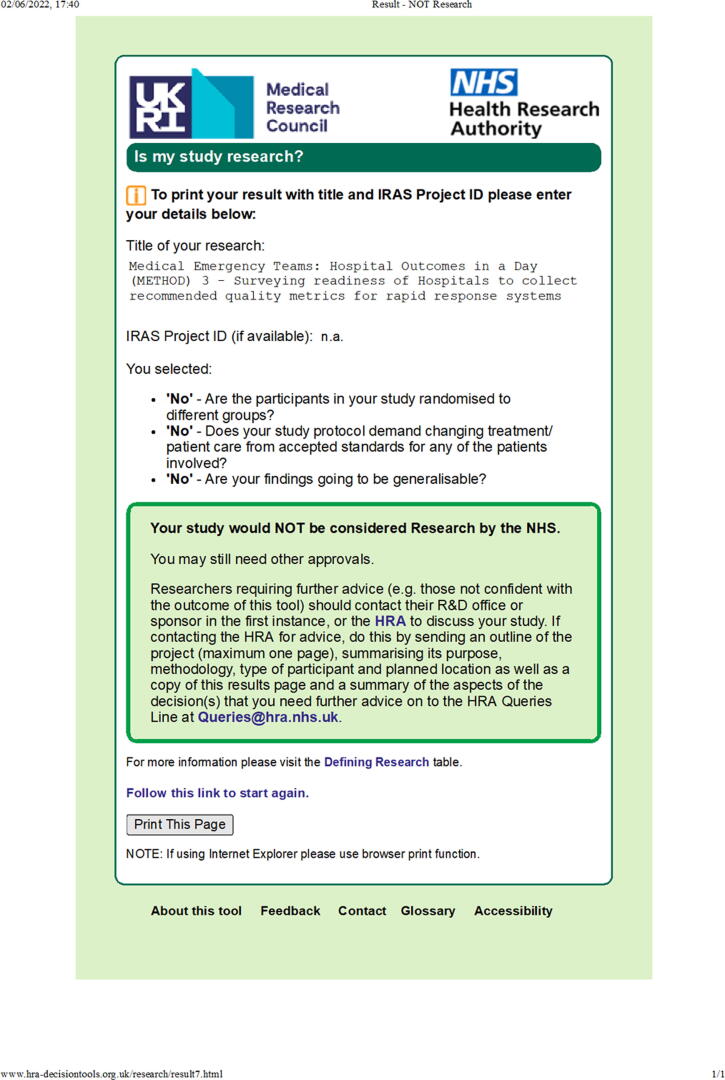# The third Medical Emergency Teams – Hospital outcomes in a day (METHOD3) study: The application of quality metrics for rapid response systems around the world

**DOI:** 10.1016/j.resplu.2023.100502

**Published:** 2023-11-11

**Authors:** Filip Haegdorens, Eirian Edwards, Ralph K. So, Christian P. Subbe

**Affiliations:** a Centre for Research and Innovation in Care (CRIC), University of Antwerp, Belgium; b Advanced Nurse Practitioner, Betsi Cadwaladr University Health Board, Bangor, Gwynedd, United Kingdom; c Intensive Care and Medical Manager Department Quality, Safety and Innovation, Albert Schweitzer Hospital, Dordrecht, The Netherlands; d Bangor University, School of Medical Sciences, Bangor, United Kingdom

**Keywords:** Rapid Response System, Medical Emergency Team, Cardiac Arrest, Quality, Safety

## Abstract

**Aim:**

This cross-sectional study aimed to assess the readiness of international hospitals to implement consensus-based quality metrics for rapid response systems (RRS) and evaluate the feasibility of collecting these metrics.

**Methods:**

A digital survey was developed and distributed to hospital administrators and clinicians worldwide. The survey captured data on the recommended quality metrics for RRS and collected information on hospital characteristics. Statistical analysis included descriptive evaluations and comparisons by country and hospital type.

**Results:**

A total of 109 hospitals from 11 countries participated in the survey. Most hospitals had some form of RRS in place, with multiple parameter track and trigger systems being commonly used. The survey revealed variations in the adoption of quality metrics among hospitals. Metrics related to patient-activated rapid response and organizational culture were collected less frequently. Geographical differences were observed, with hospitals in Australia and New Zealand demonstrating higher adoption of core quality metrics. Urban hospitals reported a lower number of recorded metrics compared to metropolitan and rural hospitals.

**Conclusion:**

The study highlights the feasibility of collecting consensus-based quality metrics for RRS in international hospitals. However, variations in data collection and adoption of specific metrics suggest potential barriers and the need for further exploration. Standardized quality metrics are crucial for effective RRS functioning and continuous improvement in patient care. Collaborative initiatives and further research are needed to overcome barriers, enhance data collection capabilities, and facilitate knowledge sharing among healthcare providers to improve the quality and safety of RRS implementation globally.

## Introduction

Rapid Response Systems (RRS) are used in acute hospitals around the world aiming to optimise care surrounding the deteriorating patient. Their goal is to detect early signs of patient deterioration, activate a specialized response team, and provide timely and appropriate care. During the last decade, the RRS concept has gained widespread recognition as a framework that guides hospital administrators and clinicians in how to organise care.^1^ While the RRS conceptual framework is well known and widely implemented, the assessment of RRS implementation effectiveness with regard to quality improvement remains vital for its success.

The sustainability of RRSs depends highly on how system issues are detected and addressed.^2^ Devita et al. introduced a data collection and process improvement component within the RRS framework aiming to continuously improve the embedding of the system within hospital structures.^3^ In this component, process and outcome metrics should be used to monitor RRS efficiency and effectiveness. In 2018, the international Society for Rapid Response Systems (iSRRS) convened a consensus conference to agree key indicators for RRS quality improvement.^4^ The consensus process used the framework of the quadruple aim of the Institute for Healthcare Improvement to commission scoping reviews of the literature.^5^ The results were discussed in three workstreams during a two day in person workshop in Manchester, results were then presented for discussion to a large audience at the 14th International Conference on Rapid Response Systems and Medical Emergency Teams in conjunction with HSJ’s Patient Safety Congress and subsequently confirmed by consensus. Metrics were classified as process, outcome and balancing measures and based on the strength of evidence as essential, optional, recommended, or experimental.

Despite the existence of these consensus-based quality metrics, it is unknown whether hospitals are currently collecting data on these indicators or if it is even feasible to collect this data. This is an essential piece of information needed to initiate regional collaboration through peer communities or breakthrough collaborative groups that strive to improve the quality and safety of patients at risk of catastrophic deterioration in the hospital.^6^ Standardised quality metrics are a fundamental part of a good functioning RRS and should be collected and reviewed frequently for these systems to function well and continuously improve.^7^ However, it remains unclear if hospitals are currently collecting data on RRS efficiency and effectiveness or if they can do so in the near future. This study aimed to assess the readiness of international hospitals to implement consensus-based quality metrics for rapid response systems.

## Material and methods

In this study a digital survey was developed and send out to hospital administrators and clinicians around the world. The survey captured the current metrics used in the data collection and process improvement component within the RRS framework against the recommended metrics of the international Society for Rapid Response Systems published in 2019.^4^

### Inclusion and exclusion criteria

The authors included hospitals who have a Rapid Response System or are in the process of setting up a Rapid Response System and caring for acutely unwell patients.

Hospitals caring only for palliative patients or specialising in post-acute care were excluded.

### Recruitment and data collection

Participating units were recruited through national societies of critical care or Critical Care Outreach, national or international conferences and through social media postings by the international Society for Rapid Response Systems (iSRRS). Data was collected through an online survey (surveymonkey.co.uk). The survey was open from the 1st of July to the 30th of November 2022.

The survey collected data on the size of the hospital (approximate bed number), location of the hospital (region), location type (i.e., metropolitan, rural, or urban), nature of the hospital (specialist/general/secondary/tertiary), nature of the current Rapid Response System, and details on the ability to collect data for each of the 10 recommended quality metrics.^4^ Response to each for the metrics contained categorical responses: recording already, could record, can’t record, haven’t got that service, not sure. We defined metropolitan as: “a multi comprised urban area with an urban core area that is highly densely populated”, urban as: “a human settlement with high-density, built-in infrastructure and environment which is created through urbanization”, and rural as: “a geographic region characterized by a relatively low population density”.

### Statistics and analysis

Statistical analysis was limited to a descriptive evaluation collating capability of participating units. The predefined analysis plan included reporting of results by country and by size and type of hospital. An online tool was used to draw a map showing the location of all participating hospitals.^8^ The characteristics of participating hospitals and the application of standardised quality metrics were compared between geographical locations using Pearson Chi-Squared tests. The number of core metrics that are currently recorded or could be recorded were combined in a newly constructed variable per hospital in the dataset (minimum 0, maximum 10). To compare the number of core metrics that are currently recorded or could be recorded between regions and location types (i.e., metropolitan, rural, urban location), a Kruskal-Wallis test was used since this test is more appropriate for variables with limited categories and potentially many ties.^9^

### Ethical considerations

The Investigators ensured that this study was conducted in accordance with the principles of the Declaration of Helsinki. Consent was not required as the survey collected no patient related data. Data collection did not include any identifiable or patient related data. Hospital names were recorded for the purpose of assuring data quality and the analysis was pseudonymised in relation to the hospital name. The author performing all analyses was blinded for the hospital name and worked only with the pseudonymised dataset. All documents will be stored securely and only accessible by study staff and authorised personnel. The study complied with the Data Protection Act, which requires data to be anonymised as soon as it is practical to do so. A classification of the study was undertaken using the tool by the English Health Research Authority (HRA) screening tool and the survey was not classified as research (Appendix 1). No expenses or benefits were provided to participating hospitals or data collectors.

## Results

**Fig. 1:**
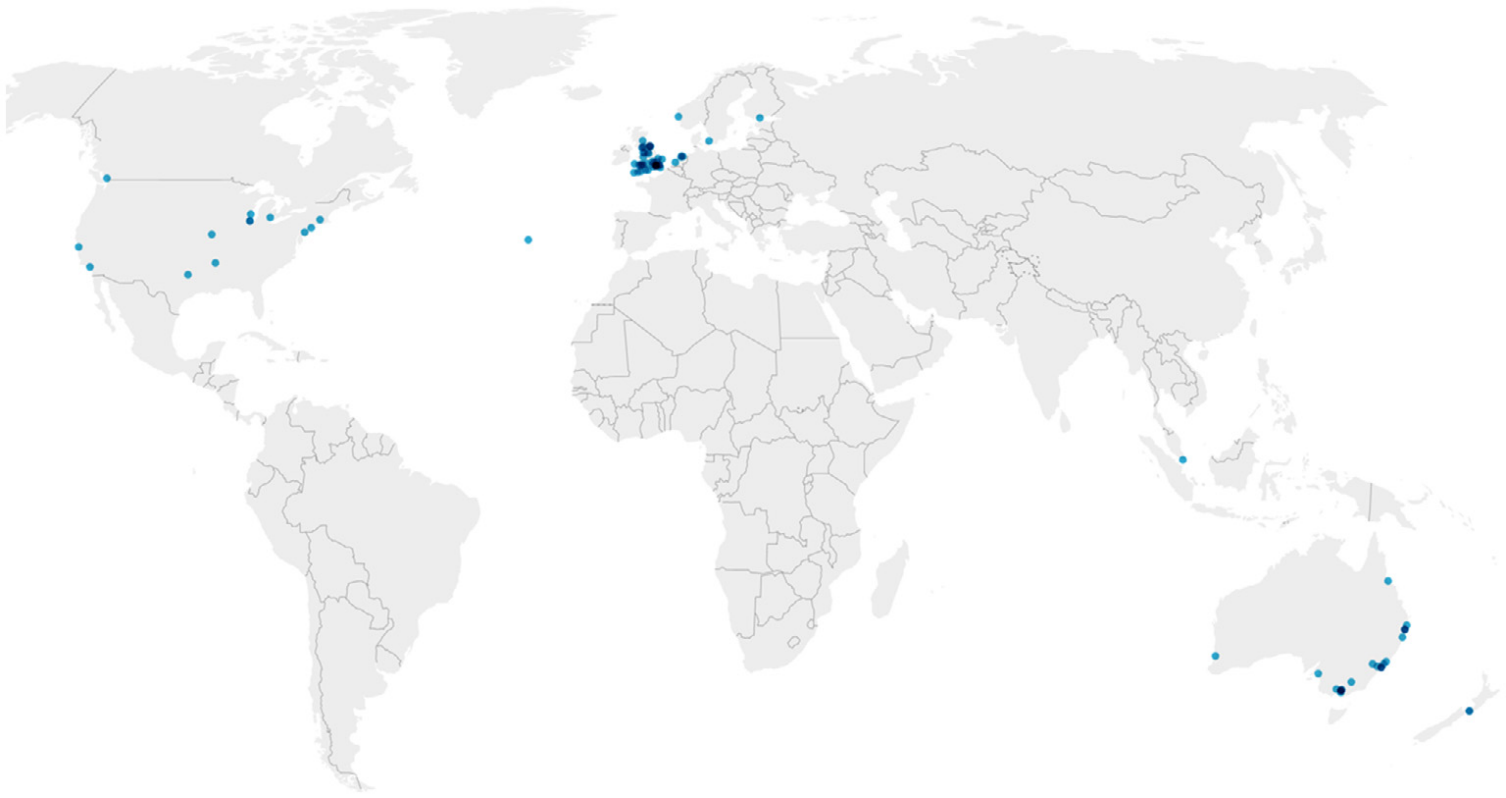
Location of participating hospitals with a known location (*n* = 92). *Created using**datawrapper.de*.

In total 109 hospitals participated in the survey. The location data was not shared by 17 hospitals. This resulted in full data of 92 hospitals located in 11 countries: Australia (*n* = 23), Canada (*n* = 1), Denmark (*n* = 1), Finland (*n* = 1), the Netherlands (*n* = 4), New Zealand (*n* = 2); Norway (*n* = 1), Portugal (*n* = 1), Singapore (*n* = 1), United Kingdom (*n* = 45), and the United States of America (*n* = 12). The exact location of each participating hospital is mapped in Fig. 1 where each dot represents a hospital. The point in the Atlantic Ocean represents a hospital on Terceira Island which is part of the Azores (Portugal). Half of all participating hospitals (57 of 109 hospitals) can be considered as large centers (i.e., >500 beds). Most of the hospitals were tertiary care centres (54 of 109 hospitals) and were located in an urban environment (urban: 52, metropolitan: 48, rural: 9 hospitals).

Almost all participants had some form of rapid response system in place (Table 1). The majority of hospitals are using a multiple parameter track and trigger system in the afferent limb and a medically led rapid response system in the efferent limb of their RRS. In the UK and Europe, multiple parameter track and trigger systems are predominantly in place while single parameter trigger systems are preferred in Australia, New Zealand and one hospital in Singapore. Surprisingly, in one in five of the participating hospitals from the USA and Canada (*n* = 3 hospitals), no afferent limb was in place. A medically led rapid response team is preferred in most of the participating hospitals around the world. However, half of the centers in the UK are using a nurse led system in the efferent limb.

**Table 1:** Characteristics of participating hospitals per geographical location.

		Afferent limb			Efferent limb			
	Have a rapid response system in place	Multiple parameters trigger	Single parameter trigger	No afferent limb	Medically led rapid response	Nurse led rapid response	Other system	No efferent limb
	%	%	%	%	%	%	%	%
Total (*n* = 109)	98.2	71.6	25.7	2.8	46.8	35.8	13.8	3.7
United Kingdom (*n* = 45)	95.6	95.6	4.4	0.0	22.2	53.3	17.8	6.7
Australia & New Zealand (*n* = 25)	100	44.0	56.0	0.0	76.0	16.0	8.0	0.0
USA & Canada (*n* = 13)	100	53.8	23.1	23.1	53.8	38.5	7.7	0.0
Europe (*n* = 8)	100	87.5	12.5	0.0	75.0	12.5	12.5	0.0
Singapore (*n* = 1)	Yes	No	Yes	No	Yes	No	No	No
*p*-value*	0.711	<0.001			0.014			

*n* = 109 hospitals participated, 17 have missing data concerning location.

* Pearson Chi-Squared tests.

**Fig. 2:**
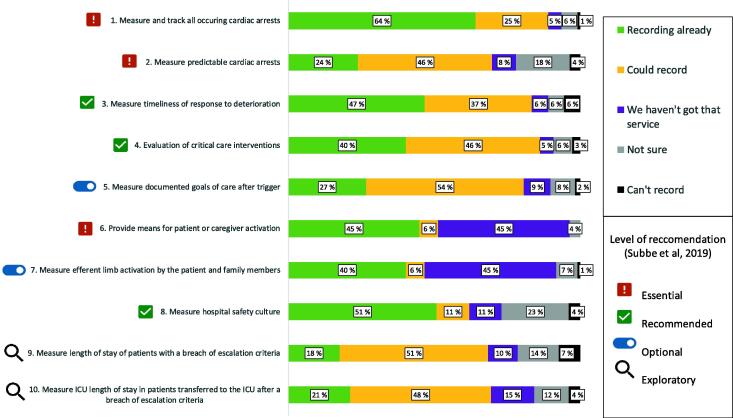
Percentages of hospitals indicating the application of the ten core metrics on the assessment of Rapid Response Systems (*n* = 109).

We asked participants to indicate if they were able to record each of the 10 core metrics on the assessment of Rapid Response Systems (Fig. 2). Most hospitals are recording cardiac arrests and measuring hospital safety culture. Between 40 and 47% of hospitals use and measure patient or caregiver efferent limb activation, evaluate critical care interventions, and measure timeliness of response to deterioration. Metrics 2, 5, 9, and 10 are least used in practice. However, most respondents indicate that they are recording or could record these metrics.

**Fig. 3:**
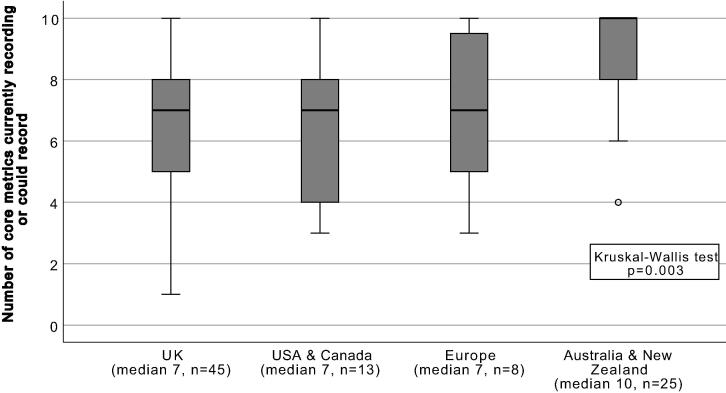
Geographic comparison of the number of core metrics that are currently recorded or could be recorded by participating hospitals (maximum is 10). *UK = United Kingdom, USA = United States of America*.

We compared the number of core metrics that participants indicated as “recording already” or “could record” between geographic locations in Fig. 3. Only one hospital indicated that they are not recording any of the ten core metrics but had an RRS in place with a multiple parameter system and a nurse led response team (the hospital’s location was unknown). Twenty-four hospitals recorded all ten metrics (i.e., 22%, *n* = 109). The median number of core metrics in the total sample was 7 with a range of 0–10. Australia and New Zealand showed a significantly higher adoption of the core quality metrics for the evaluation of Rapid Response Systems compared with other regions (median of 10 versus 7 in other locations, Kruskal-Wallis *p* = 0.003).

When comparing each core metric, we found that metric 6 (provide means for caregiver activation), metric 7 (measure efferent limb activation by the patient and family members), and metric 8 (measure hospital safety culture) were recorded the least in the UK (Table 2). Additionally, metric 5 (measuring documented goals of care after trigger) was least measured in Europe in comparison with the other locations.

**Table 2:** Geographic comparison per core metric of hospitals indicating that they are already recording or could record.

	Metric 1	Metric 2	Metric 3	Metric 4	Metric 5	Metric 6	Metric 7	Metric 8	Metric 9	Metric 10
United Kingdom (*n* = 45)	89%	76%	82%	84%	82%	29%	24%	47%	71%	67%
Australia & New Zealand (*n* = 25)	100%	88%	88%	96%	96%	84%	80%	80%	76%	76%
USA & Canada (*n* = 13)	77%	54%	77%	85%	77%	62%	46%	77%	46%	54%
Europe (*n* = 8)	88%	75%	100%	75%	50%	50%	38%	75%	75%	75%
Singapore (*n* = 1)	Yes	Yes	Yes	Yes	Yes	No	No	Yes	Yes	Yes
*p*-value	0.233	0.214	0.615	0.513	0.049	<0.001	<0.001	0.034	0.348	0.621

Comparison between countries per metric using the Pearson Chi Squared test.

**Fig. 4:**
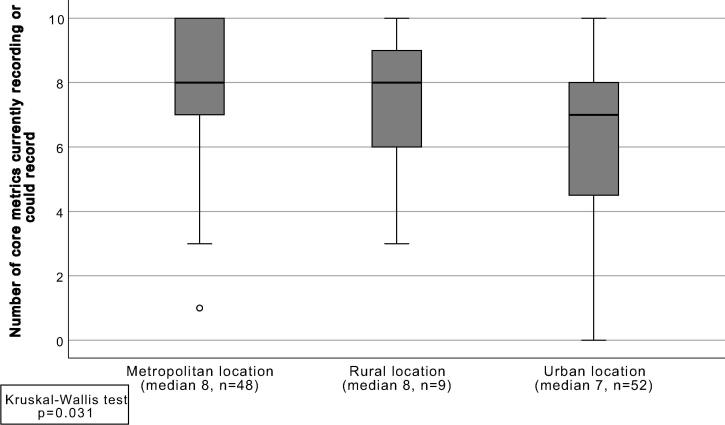
Comparison of the number of core metrics that is currently recorded or could be recorded by participating hospitals between location types (maximum is 10).

Not only the geographical location but the area where the hospital was located influenced the adoption of the core quality metrics for the evaluation of Rapid Response Systems (Fig. 4). Hospitals located in an urban setting indicated a lower number of recorded metrics compared with metropolitan or rural settings (respectively medians of 7, 8 and 8).

## Discussion

### What we have found

Hospitals indicated that they were able to collect the majority of the recommended quality indicators. The metrics by the Society for Rapid Response Systems are therefore feasible as a framework for quality improvement. Quality indicators 6 to 8 which asked about Patient Activated Rapid Response and surveys of organisational culture and capability were collected less often. Those indicators have in common that they are linked to organisational culture and require dedicated systems for data collection which might not be available in all hospitals. Patient or family activated rapid response is, at this moment in time, not yet common practice. There are studies in Australia and the USA supporting patient or family activated rapid response within the RRS framework aiming to improve patient and family participation in care and to increase safety.^10^, ^11^, ^12^ However, implementing patient or family activated rapid response is not without challenges such as barriers for activation and training rapid response team members to handle patient or family calls.^13^, ^14^, ^15^

In Australia and New-Zealand the highest number of quality metrics were being used compared with other parts of the world. One of the reasons could be that RRSs have been present in Australian and New-Zealand for more than 20 years.^16^, ^17^ Additionally, the National Safety and Quality Health Service Standard 9 of the Australian Commission on Safety and Quality in Health Care provides National guidance on how to install and maintain RRSs in acute hospitals.^18^

A difference was found in the number of quality metrics that hospitals record between the location type of hospitals. Urban hospitals reported a lower number of metrics compared with rural and metropolitan hospitals. We included only nine hospitals that were located in a rural setting and therefore these numbers could be not representative. However, for metropolitan hospitals and urban hospitals we achieved an adequate sample size. In previous research, an association was found between hospital location (urban vs. rural) and patient safety outcomes.^19^ Additionally, hospitals in urban settings have greater access to information systems, functional applications and technological devices compared with rural hospitals.^20^ We hypothesise that hospitals in metropolitan settings have more means to collect data on patient safety in a continuous manner. However, more research is needed to confirm or reject this hypothesis.

In one in five hospitals from the USA and Canada, no afferent limb was in place. Traditionally, RRSs in the United States are focused on the efferent limb (e.g., the rapid response team or medical emergency team) thus an Early Warning Score or other multiple parameter track and trigger system are not always used in practice.^21^ This could be due to the experienced uncertainty on what thresholds to use in track-and-trigger scores to limit false-positives and how to combine a score based on vital signs with a nurse’s sense of worry.^22^ In the United States, staff worry was the specific trigger in a quarter of all rapid response team calls.^21^

### Strength and weaknesses

This is the first international survey of its kind. The recruitment was through specialist societies, and this might have selected enthusiastic early adopters of Rapid Response Systems and limited the number of hospitals that would have been aware of the survey. It might have also limited generalisability to hospitals and countries with less mature systems.^23^, ^24^, ^25^

There was a clear hierarchy in the frequency of responses from different countries with the United Kingdom having the highest number of participants. Extrapolation of data to the situation in a whole country is therefore limited. Another important limitation is that we did not include any other Asian countries than Singapore where RRSs are currently in place. In future studies on this topic, researchers should attempt to include hospitals from all geographic regions around the world.

We asked units to indicate core metrics that they are currently recording or could record, however we are unsure how this data is used to drive quality. Lastly, it is essential to acknowledge potential sources of bias inherent in the use of self-administered surveys. Hospital characteristics and rapid response system composition were part of the questionnaire and could have been inaccurately answered.

### Clinical implications

Given that the collection of data for the metric seems to be feasible, hospital networks or break-through collaboratives could use the framework to drive quality for organisations or help clinicians to share data and learning for improvement. The geographic differences in reporting of patient activated rapid response and organisational culture would appear to be driven by health care policy. It is therefore of crucial importance to engage regional and national governments in the assurance process.

### Research implications

The data on metrics for patient-activated-rapid-response and safety culture might require more context to understand why hospitals don’t have access to this data or are not collecting it. It will require are more detailed understanding barriers and enablers for wider spread of high-quality care. How units can collaborate to improve quality remains to be explored further.

## Conclusions

Our findings indicate that most hospitals can collect recommended quality indicators, suggesting the feasibility of using these metrics for quality improvement in rapid response systems. However, certain metrics related to patient-activated rapid response and organizational culture were collected less frequently, highlighting potential barriers and the need for further investigation. Geographic variations and differences based on hospital location type were also found and could be due to limited access to information technology in non-metropolitan hospitals. Overall, a standardized set of quality metrics are essential for effective RRS functioning and continuous improvement in patient care.

## Funding

None.

## CRediT authorship contribution statement

**Filip Haegdorens:** Formal analysis, Investigation, Resources, Data curation, Writing – original draft, Writing – review & editing, Visualization. **Eirian Edwards:** Conceptualization, Methodology, Investigation, Data curation, Writing – review & editing, Project administration. **Ralph K. So:** Conceptualization, Methodology, Writing – original draft, Writing – review & editing. **Christian P. Subbe:** Conceptualization, Methodology, Investigation, Data curation, Writing – original draft, Writing – review & editing, Visualization, Supervision.

## Declaration of competing interest

The authors declare that they have no known competing financial interests or personal relationships that could have appeared to influence the work reported in this paper.

## Figures and Tables

**Figure:**